# Symptoms of Anxiety or Depressive Disorder and Use of Mental Health Care Among Adults During the COVID-19 Pandemic — United States, August 2020–February 2021

**DOI:** 10.15585/mmwr.mm7013e2

**Published:** 2021-04-02

**Authors:** Anjel Vahratian, Stephen J. Blumberg, Emily P. Terlizzi, Jeannine S. Schiller

**Affiliations:** 1 National Center for Health Statistics, CDC.

The spread of disease and increase in deaths during large outbreaks of transmissible diseases is often associated with fear and grief (*1*). Social restrictions, limits on operating nonessential businesses, and other measures to reduce pandemic-related mortality and morbidity can lead to isolation and unemployment or underemployment, further increasing the risk for mental health problems (*2*). To rapidly monitor changes in mental health status and access to care during the COVID-19 pandemic, CDC partnered with the U.S. Census Bureau to conduct the Household Pulse Survey (HPS). This report describes trends in the percentage of adults with symptoms of an anxiety disorder or a depressive disorder and those who sought mental health services. During August 19, 2020–February 1, 2021, the percentage of adults with symptoms of an anxiety or a depressive disorder during the past 7 days increased significantly (from 36.4% to 41.5%), as did the percentage reporting that they needed but did not receive mental health counseling or therapy during the past 4 weeks (from 9.2% to 11.7%). Increases were largest among adults aged 18–29 years and among those with less than a high school education. HPS data can be used in near real time to evaluate the impact of strategies that address mental health status and care of adults during the COVID-19 pandemic and to guide interventions for groups that are disproportionately affected.

HPS is a rapid-response online survey using a probability-based sample design to measure the social and economic impact of the COVID-19 pandemic on U.S. households (*3*). This experimental data product* was developed by the U.S. Census Bureau in partnership with CDC’s National Center for Health Statistics (NCHS) and several other federal statistical agencies. The sample is drawn from the U.S. Census Bureau’s Master Address File.^†^ E-mail addresses and mobile telephone numbers associated with randomly selected housing units are used to invite participants. Analytic files include self-reported data from one adult aged ≥18 years at each address. Data collection began on April 23, 2020, and is ongoing (phase 1 = April 23–July 21, 2020; phase 2 = August 19–October 26, 2020; phase 3 = October 28, 2020–present, with a break during December 22, 2020–January 5, 2021). HPS response rates averaged 2.9%, 9.3%, and 6.5% during phase 1, phase 2, and phase 3 (through February 1, 2021), respectively.

Questions on mental health symptoms were based on the validated four-item Patient Health Questionnaire (PHQ-4) for depression and anxiety and included how often, during the past 7 days, respondents had been bothered by 1) feeling nervous, anxious, or on edge; 2) not being able to stop or control worrying; 3) having little interest or pleasure in doing things; and 4) feeling down, depressed, or hopeless. Adults who had symptoms that generally occurred more than one half of the days or nearly every day were classified as having symptoms, consistent with published scoring recommendations^§^ (*4*). Questions about mental health care use included whether, during the past 4 weeks, respondents had 1) taken prescription medication for their mental health, 2) received counseling or therapy from a mental health professional, or 3) needed but did not receive counseling or therapy from a mental health professional (i.e., had an unmet mental health need).

Because of methodological differences between phases 1 and 2, trend analyses were limited to phases 2 and 3.^¶^ Estimates** are presented for each 2-week data collection period for August 19, 2020–February 1, 2021 (unweighted sample size = 431,656 for phase 2 and 358,977 for phase 3, total = 790,633). Trends were assessed using joinpoint regression.^††^ In addition, changes in estimates of symptoms of an anxiety or a depressive disorder and the two mental health care measures were compared between August 19–31, 2020, and January 20–February 1, 2021, according to selected respondent characteristics. SAS-callable SUDAAN (version 11.0; RTI International) was used to conduct these analyses. Estimates were weighted to adjust for nonresponse and number of adults in the household and to match U.S. Census Bureau estimates of the population by age, sex, race/ethnicity, and educational attainment.

During August 19–31, 2020, through December 9–21, 2020, significant increases were observed in the percentages of adults who reported experiencing symptoms of an anxiety disorder (from 31.4% to 36.9%), depressive disorder (from 24.5% to 30.2%), and at least one of these disorders (from 36.4% to 42.4%) (Figure 1). Estimates for all three mental health indicators through January 2021 were similar to those in December 2020.

**FIGURE 1:**
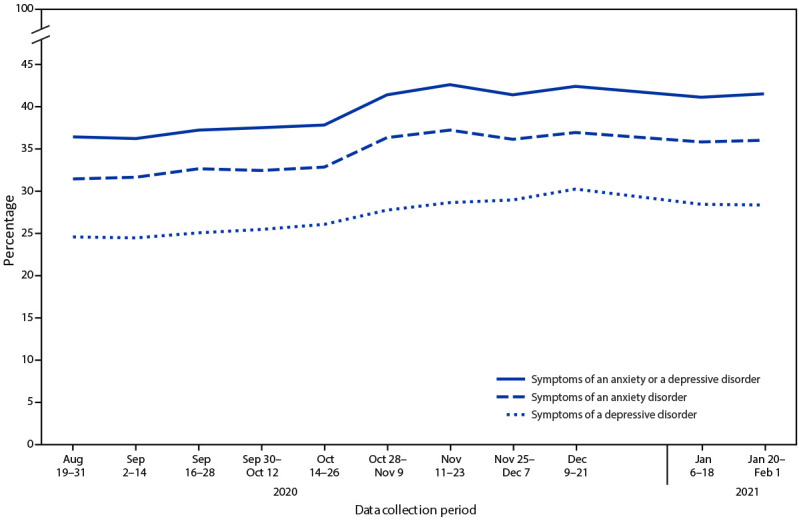
Percentage of adults aged ≥18 years with symptoms of anxiety disorder, depressive disorder, or anxiety or depressive disorder during past 7 days, by data collection period — Household Pulse Survey, United States, August 19, 2020–February 1, 2021* * Household Pulse Survey data collection included a 1-day break between the conclusion of one data collection period and the start of the next, as well as a 2-week break during December 22, 2020–January 5, 2021.

During August 19–31, 2020, through November 25–December 7, 2020, a significant increase was observed in the percentage of adults who reported taking prescription medication or receiving counseling for their mental health (from 22.4% to 25.0%) (Figure 2). Similarly, during August 19–31, 2020, through December 9–21, 2020, a significant increase was observed in the percentage of adults who reported needing but not receiving counseling or therapy for their mental health (from 9.2% to 12.4%). Estimates through January 2021 were similar to those in December 2020.

**FIGURE 2:**
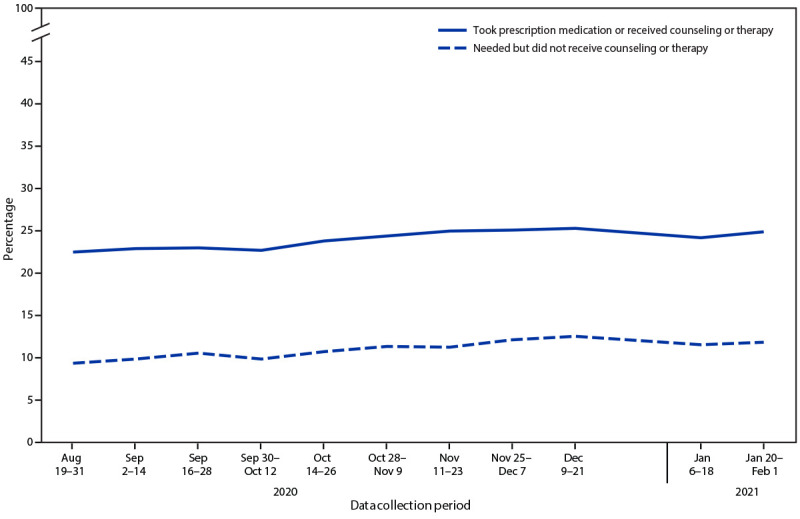
Percentage of adults aged ≥18 years who took prescription medication for mental health or received counseling or therapy during past 4 weeks and percentage who needed but did not receive counseling or therapy during past 4 weeks, by data collection period — Household Pulse Survey, United States, August 19, 2020–February 1, 2021* * Household Pulse Survey data collection included a 1-day break between the conclusion of one data collection period and the start of the next, as well as a 2-week break during December 22, 2020–January 5, 2021.

During August 19–31, 2020, through January 20–February 1, 2021, symptoms of an anxiety or a depressive disorder increased significantly from 36.4% to 41.5% (Table). Significant increases were observed for all demographic subgroups presented, except adults aged ≥80 years and non-Hispanic adults reporting races other than White, Black, or Asian. The largest increases (8.0 and 7.8 percentage points) were among those aged 18–29 years and those with less than a high school education, respectively. During this time, mental health care treatment increased significantly from 22.4% to 24.8%. Significant increases were observed for adults aged 18–29, 30–39, and 60–69 years; men and women; non-Hispanic White and non-Hispanic Black adults; adults with at least a high school education; and adults who had not experienced symptoms of an anxiety or a depressive disorder during the past 7 days. 

**TABLE:** Weighted* percentage of adults aged ≥18 years with symptoms of anxiety or depressive disorder during past 7 days, percentage who took prescription medication for mental health or received counseling or therapy during past 4 weeks, and percentage who needed but did not receive counseling or therapy during past 4 weeks, by selected characteristics — Household Pulse Survey, United States, August 19, 2020–February 1, 2021

Characteristic	% (95% CI)					
	Symptoms of anxiety or depressive disorder during past 7 days		Took prescription medication for mental health or received counseling or therapy during past 4 weeks		Needed but did not receive counseling or therapy during past 4 weeks	
	Aug 19–31, 2020	Jan 20–Feb 1, 2021	Aug 19–31, 2020	Jan 20–Feb 1, 2021	Aug 19–31, 2020	Jan 20–Feb 1, 2021
**Total**	**36.4 (35.9–36.9)**	**41.5 (40.7–42.2)^†^**	**22.4 (22.0–22.9)**	**24.8 (24.2–25.4)^†^**	**9.2 (8.8–9.6)**	**11.7 (11.1–12.2)^†^**
**Age group, yrs**						
18–29	49.0 (47.5–50.5)	57.0 (54.2–59.8)^†^	23.3 (21.5–25.2)	26.9 (24.9–29.0)^†^	15.6 (14.5–16.7)	22.8 (20.3–25.4)^†^
30–39	42.5 (40.8–44.1)	45.9 (44.5–47.3)^†^	23.1 (22.1–24.1)	27.1 (25.8–28.4)^†^	12.9 (11.9–13.9)	16.1 (14.8–17.5)^†^
40–49	37.6 (36.3–39.0)	41.1 (38.9–43.2)^†^	23.6 (22.8–24.5)	25.0 (23.7–26.3)	10.0 (9.3–10.7)	11.0 (10.0–11.9)
50–59	34.9 (33.6–36.3)	41.2 (39.8–42.6)^†^	23.9 (22.8–25.1)	25.4 (24.0–26.9)	7.7 (6.9–8.5)	9.5 (8.6–10.4)^†^
60–69	29.3 (28.0–30.6)	33.4 (31.6–35.4)^†^	21.2 (20.2–22.2)	23.3 (22.0–24.6)^†^	5.3 (4.8–5.9)	5.4 (4.8–6.0)
70–79	23.2 (21.6–25.0)	26.3 (24.6–28.0)^†^	19.6 (18.1–21.1)	19.8 (18.3–21.3)	2.9 (2.2–3.6)	3.1 (2.4–3.9)
≥80	19.4 (16.3–22.9)	22.5 (18.5–27.0)	14.8 (12.0–17.9)	17.3 (14.1–21.0)	1.4 (0.9–2.0)	2.3 (1.3–3.7)
**Sex**						
Male	31.8 (30.8–32.8)	38.0 (36.9–39.1)^†^	16.3 (15.6–17.1)	19.1 (18.1–20.1)^†^	6.8 (6.2–7.3)	9.1 (8.3–9.8)^†^
Female	40.7 (39.9–41.5)	44.8 (43.8–45.8)^†^	28.0 (27.3–28.7)	30.0 (29.3–30.7)^†^	11.4 (10.9–11.9)	14.1 (13.4–14.8)^†^
**Race/Ethnicity**						
Hispanic or Latino	40.2 (38.0–42.3)	47.1 (44.7–49.4)^†^	17.2 (15.8–18.6)	19.5 (17.3–21.9)	9.6 (8.6–10.6)	12.8 (10.9–14.9)^†^
White, non-Hispanic	35.4 (34.8–35.9)	39.8 (38.9–40.7)^†^	25.6 (25.0–26.1)	28.1 (27.3–28.8)^†^	9.1 (8.7–9.5)	11.7 (11.2–12.1)^†^
Black, non-Hispanic	37.7 (35.7–39.8)	44.5 (41.6–47.5)^†^	15.6 (14.2–17.1)	18.7 (16.7–20.8)^†^	9.3 (8.3–10.3)	12.2 (10.4–14.1)^†^
Asian, non-Hispanic	30.5 (28.2–32.8)	37.4 (33.4–41.5)^†^	11.1 (9.7–12.5)	12.9 (10.7–15.4)	4.8 (3.9–5.8)	5.8 (4.5–7.3)
Other/Multiple races, non-Hispanic	43.1 (40.2–46.1)	44.8 (41.0–48.6)	25.0 (22.3–27.9)	23.8 (20.9–26.9)	14.2 (12.1–16.4)	13.8 (11.4–16.5)
**Education level**						
Less than high school diploma	41.8 (38.4–45.2)	49.6 (45.7–53.5)^†^	20.0 (17.3–22.9)	20.6 (17.5–24.0)	7.0 (5.4–8.8)	11.3 (8.8–14.2)^†^
High school diploma or GED certificate	36.3 (35.0–37.7)	41.1 (39.3–42.9)^†^	20.1 (19.1–21.2)	22.2 (20.9–23.4)^†^	7.0 (6.3–7.8)	8.7 (7.4–10.2)^†^
Some college or associate’s degree	39.4 (38.5–40.3)	46.4 (45.2–47.6)^†^	23.5 (22.7–24.4)	27.7 (26.8–28.7)^†^	11.2 (10.6–11.9)	14.9 (13.9–15.9)^†^
Bachelor’s degree or higher	32.4 (31.7–33.0)	35.5 (34.7–36.3)^†^	24.0 (23.4–24.6)	25.4 (24.6–26.1)^†^	9.7 (9.2–10.1)	11.4 (10.9–12.0)^†^
**Symptoms of anxiety or depressive disorder during past 7 days**						
Did not experience symptoms	NA	NA	13.9 (13.4–14.4)	15.6 (14.9–16.4)^†^	2.4 (2.2–2.7)	3.1 (2.8–3.5)^†^
Experienced symptoms	NA	NA	37.5 (36.5–38.5)	37.7 (36.6–38.8)	21.0 (20.2–21.8)	23.8 (22.8–24.9)^†^

**Abbreviations:** CI = confidence interval; GED = general educational development; NA = not applicable.

* Estimates were weighted to adjust for nonresponse and number of adults in the household and to match U.S. Census Bureau estimates of the population by age, sex, race/ethnicity, and educational attainment.

^†^ Significant difference between percentages at two time points (August 19–31, 2020, versus January 20–February 1, 2021) based on two-sided significance tests at the 0.05 level.

Unmet mental health needs also increased significantly from 9.2% to 11.7%. Subgroups with significant increases included adults aged 18–29, 30–39, and 50–59 years; men and women; Hispanic, non-Hispanic White, and non-Hispanic Black adults; adults at all education levels; and adults regardless of whether they experienced symptoms of an anxiety or a depressive disorder or both during the past 7 days. The largest increases in unmet mental health needs (7.2 percentage points and 4.3 percentage points) were among adults aged 18–29 years and those with less than a high school education, respectively. During January 20, 2021–February 1, 2021, 23.8% of persons with symptoms of an anxiety or a depressive disorder had unmet mental health needs, and this percentage increased by 2.8 percentage points from August 2020 to February 2021. 

## Discussion

The percentage of adults who had symptoms of an anxiety or a depressive disorder during the past 7 days and those with unmet mental health needs during the past 4 weeks increased significantly from August 2020 to February 2021, with the largest increases among those aged 18–29 years and those with less than a high school education. During January 20, 2021–February 1, 2021, more than two in five adults aged ≥18 years experienced symptoms of an anxiety or a depressive disorder during the past 7 days. One in four adults who experienced these symptoms reported that they needed but did not receive counseling or therapy for their mental health.

These findings are consistent with results from surveys conducted early in the COVID-19 pandemic (March–June 2020) that showed an increased prevalence of mental health symptoms, especially among young adults (*5*–*7*). The more recent results indicate an increasing prevalence over time later in 2020, which remained increased in early 2021. The trends in symptoms of an anxiety or a depressive disorder from HPS have been shown to be consistent with trends in the weekly number of reported COVID-19 cases, and it has been theorized that increases in these mental health indicators correspond with pandemic trends (*8*).

The findings in this report are subject to at least four limitations. First, these data are based on self-report and were not confirmed by health professionals. Questions about mental health symptoms might be predictive of but do not necessarily reflect a clinical diagnosis. In addition, the predictive validity of the scales used in this report have not been confirmed when adapted from a 2-week to a 1-week time frame. Second, HPS did not assess the cause of these symptoms; therefore, a direct association with COVID-19 events could not be determined with certainty. Third, changes in mental health symptoms from the summer to the winter months might reflect symptoms associated with seasonal affective disorder (*9*). However, data from the 2019 National Health Interview Survey (NHIS),^§§^ measured using the unmodified PHQ-4, did not demonstrate statistically significant changes from August to December 2019 for symptoms of an anxiety disorder (8.1% to 8.6%); a depressive disorder (7.0% to 6.7%); or an anxiety disorder, a depressive disorder, or both (11.0% to 11.3%) (*10*). Finally, these estimates are intended to represent all adults aged ≥18 years living in housing units in the United States. However, representativeness might be limited by the indirect exclusion of persons without Internet access and by low response rates. Some households were not eligible to participate because the U.S. Census Bureau was unable to match a mobile telephone number or e-mail address. The sampling weights that were applied to all analyses were likely to have reduced some of the potential bias. Nevertheless, these data might not fully meet the U.S. Census Bureau’s quality standards and as such, the bureau labeled these data as experimental.

Despite these limitations, the survey’s timeliness and relevance are strengths of HPS. The U.S. Census Bureau releases data tables to the public 9 days after the close of each data collection period.^¶¶^ Simultaneously, NCHS updates online visualizations of trends in key health indicators.***

Several measures have been initiated to address increased mental health risks associated with COVID-19,^†††^ and a previous report outlines additional strategies, including expanded use of telehealth, to address mental health conditions during the pandemic (*6*). Continued near real-time monitoring of mental health trends by demographic characteristics is critical during the COVID-19 pandemic. These trends might be used to evaluate the impact of strategies that address mental health status and care of adults during the pandemic and to guide interventions for groups that are disproportionately affected.

**Summary**

**What is already known about this topic?**

Large disease outbreaks have been associated with mental health problems.

**What is added by this report?**

During August 2020–February 2021, the percentage of adults with recent symptoms of an anxiety or a depressive disorder increased from 36.4% to 41.5%, and the percentage of those reporting an unmet mental health care need increased from 9.2% to 11.7%. Increases were largest among adults aged 18–29 years and those with less than a high school education.

**What are the implications for public health practice?**

Trends in mental health can be used to evaluate the impact of strategies addressing adult mental health status and care during the pandemic and to guide interventions for disproportionately affected groups.

## Footnotes

* U.S. Census Bureau experimental data products are statistical products created using new data sources or previously untested methodologies. The analysis in this report was based on publicly available deidentified data files provided by the U.S. Census Bureau. https://www.census.gov/data/experimental-data-products.html

† The Master Address File is the U.S. Census Bureau’s official inventory of known living quarters in the United States, maintained to facilitate the decennial census. E-mail addresses, mobile telephone numbers, or both were appended for 81% of addresses. Housing units linked to one or more e-mail addresses or mobile telephone numbers are randomly selected to participate.

§ These items are adapted from the validated PHQ-4 for depression and anxiety, which includes the two-item versions of the Generalized Anxiety Disorder (GAD-2, items 1 and 2) scale and the two-item PHQ (PHQ-2, items 3 and 4). Because phase 1 of HPS was conducted weekly, the recall period for these questions was modified from a 2-week to a 1-week period for this survey. For each survey response, answers were assigned a numerical value: not at all (0), several days (1), more than one half of the days (2), and nearly every day (3). The two responses for anxiety symptoms were summed, and adults who had scale scores of ≥3 were classified as having symptoms of an anxiety disorder. The two responses for depressive symptoms were summed, and adults who had scale scores of ≥3 were classified as having symptoms of a depressive disorder. These two composite indicators were used to create the third composite indicator of symptoms of an anxiety disorder, a depressive disorder, or both. This scoring approach is the same as recommended by the developers of PHQ-2 and GAD-2.

¶ Methodological differences between phase 1 and phase 2 complicate examination of trends across the two phases. These differences include a change in the data collection period from 6 days to 13 days, additional reminders sent to nonrespondents in phase 2, and elimination of a longitudinal component that was present in phase 1. Therefore, trends are only examined for phases 2 and 3. Sample sizes for the mental health questions averaged 86,000 completed surveys biweekly in phase 2 and 60,000 biweekly in phase 3 through February 1, 2021.

** All estimates shown met the NCHS Data Presentation Standards for Proportions (https://www.cdc.gov/nchs/data/series/sr_02/sr02_175.pdf).

†† Joinpoint regression characterizes trends as joined linear segments (https://surveillance.cancer.gov/joinpoint/). A joinpoint is the period at which two segments with different slopes meet. Joinpoint software uses statistical criteria to determine the fewest number of segments necessary to characterize a trend and the periods when segments begin and end. The models were specified to include a minimum of one joinpoint.

§§ NHIS is an annual household survey of the noninstitutionalized U.S. civilian population. In 2019, NHIS included the eight-item PHQ (PHQ-8) depression scale and seven-item GAD (GAD-7) scale as part of its sample adult interview.

¶¶ The U.S. Census Bureau releases data files to the public within 4 weeks after the close of each data collection period. https://www.census.gov/programs-surveys/household-pulse-survey/data.html

***
https://www.cdc.gov/nchs/covid19/health-care-access-and-mental-health.htm

††† https://howrightnow.org/; https://www.cdc.gov/coronavirus/2019-ncov/daily-life-coping/managing-stress-anxiety.html; https://www.samhsa.gov/find-help/disaster-distress-helpline
